# Enhanced Prevalence of Plasmatic Soluble MHC Class I Chain-Related Molecule in Vascular Pregnancy Diseases

**DOI:** 10.1155/2014/653161

**Published:** 2014-08-27

**Authors:** Jean Baptiste Haumonte, Sophie Caillat-Zucman, Florence Bretelle, Marion Lambert, Luc Lyonnet, Annie Levy-Mozziconacci, Catherine Farnarier, Agostini Aubert, Leon Boubli, Laurence Camoin-Jau, Françoise Dignat George, Pascale Paul

**Affiliations:** ^1^Assistance Publique-Hôpitaux de Marseille, Aix Marseille Université, Department of Gynecology and Obstetrics, Gynépole Marseille, Hôpital Nord, Chemin des Bourrely, 13915 Marseille, France; ^2^Aix-Marseille Université, UMR 1076 INSERM, Vascular Research Center of Marseille, 3005 Marseille, France; ^3^Assistance Publique Hôpitaux de Paris, Immunology Department, Hôpital Robert Debré UMR 1149 INSERM-Université Paris Diderot, 75019 Paris, France; ^4^Assistance Publique Hôpitaux de Marseille, Hematology Department, Secteur de Biologie Vasculaire, Hôpital Conception, 13005 Marseille, France; ^5^Assistance Publique-Hôpitaux de Marseille, Aix Marseille Université Materno-fœtal Biology Unit, Secteur Nord, 13916 Marseille, France; ^6^Assistance Publique Hôpitaux de Marseille, Immunology Department, Hôpital de la Conception, 13005 Marseille, France; ^7^Assistance Publique-Hôpitaux de Marseille, Department of Gynecology and Obstetrics, Hôpital Conception, 13005 Marseille, France; ^8^Assistance Publique Hôpitaux de Marseille, Hematology Department, Hôpital Timone, 13005 Marseille, France

## Abstract

The major histocompatibility complex class I related chain (MIC) is a stress-inducible protein modulating the function of immune natural killer (NK) cells, a major leukocyte subset involved in proper trophoblast invasion and spiral artery remodeling. Aim of the study was to evaluate whether upregulation of soluble MIC (sMIC) may reflect immune disorders associated to vascular pregnancy diseases (VPD). sMIC was more frequently detected in the plasma of women with a diagnostic of VPD (32%) than in normal term-matched pregnancies (1.6%, *P* < 0.0001), with highest prevalence in intrauterine fetal death (IUDF, 44%) and vascular intrauterine growth restriction (IUGR, 39%). sMIC levels were higher in preeclampsia (PE) than in IUFD (*P* < 0.01) and vascular IUGR (*P* < 0.05). sMIC detection was associated with bilateral early diastolic uterine notches (*P* = 0.037), thrombocytopenia (*P* = 0.03), and high proteinuria (*P* = 0.03) in PE and with the vascular etiology of IUGR (*P* = 0.0038). Incubation of sMIC-positive PE plasma resulted in downregulation of NKG2D expression and NK cell-mediated IFN-*γ* production in vitro. Our work thus suggests that detection of sMIC molecule in maternal plasma may constitute a hallmark of altered maternal immune functions that contributes to vascular disorders that complicate pregnancy, notably by impairing NK-cell mediated production of IFN-*γ*, an essential cytokine favoring vascular modeling.

## 1. Introduction 

Pregnancy is a unique situation in which angiogenesis and the establishment of maternal-fetal tolerance are essential steps to ensure placental and fetal development.

Inadequate placentation leads to a large spectrum of vascular pregnancy diseases (VPD) that affects the mother and/or the fetus and include preeclampsia (PE) intrauterine fetal growth retardation (IUGR) and intrauterine fetal death (IUFD).

VPD represents a leading cause of fetomaternal morbidity and mortality. The etiology of VPD is often unknown and the search for molecules that may better characterize the physiopathological processes that lead to their adverse outcome is the subject of extensive investigation. Identification of reliable indicators of adverse outcome is a clinically relevant issue to optimize prevention of severe pregnancy complications [1], and several reports highlight the importance of both angiogenic factors in insuring adequate placentation [2–4]. Indeed, circulating antiangiogenic proteins such as soluble fms-like tyrosine kinase 1 ([sFlt1]) and soluble endoglin have been associated with the pathogenesis of PE [5, 6]. Enhanced serum levels of PAPP-A, ADMA, homocysteine, and sFlt-1 have also been associated with the severity of this disease. Despite recent advances in understanding how inadequate placental vascularization and incomplete spiral artery remodeling may lead to vascular pregnancy diseases, the prognosis of VPD often remains severe, and placental removal remains the only treatment to manage severe preeclampsia and major intrauterine growth restriction. Understanding the complex and multifactorial mechanisms that favor these severe complications is thus essential to design new therapeutic approaches. Cross-talk between fetal and maternal immune cells is an essential requisite to achieve early pregnancy placental development, vasculogenesis, and immune tolerance of the fetus [7–9]. Natural killer immune cells (NK) are a major source of angiogenic growth factors and cytokines that insure transformation of the uterine spiral arteries, fetal implantation, and placentation [2, 7, 10–12]. Abnormal NK cell receptor and cytokine production profiles have been associated with pregnancy disorders such as recurrent pregnancy loss, implantation failure, and preeclampsia [13–15]. IFN-*γ* secretion by NK cells has been identified as an essential regulatory pathway favoring vascular modeling and first trimester extravillous cytotrophoblast migration. Lowered levels of IFN-*γ* were observed in decidual NK cells from women with hypertensive disorders complicating pregnancy. In contrast, enhanced peritoneal NK cell mediated IFN-*γ* was observed in women with severe endometriosis which may promote abnormal proliferation and angiogenesis of endometrial cells [16, 17]. NK cell-mediated cytotoxic activity and angiogenic factor/cytokine production are regulated by the integration of signals that target inhibitory and activating receptors expressed on NK cells [18–23].

Various molecules have been shown to regulate uterine NK (uNK) cell-mediated transformation of decidual arteries, thereby allowing increase of placental blood supply.

Apart from its role in protecting fetal cells from maternal NK cell cytotoxicity, engagement of the KIR2DL4 receptor by HLA-G has been shown to stimulate NK-cell mediated IFN-*γ* production, thus providing a favorable environment promoting vascularization in maternal decidua during early pregnancy [24, 25]. Accordingly, lowered HLA-G expression was associated with the occurrence of preeclampsia and intrauterine growth retardation [26–29]. Engagement of NKG2D receptor by stress-inducible membrane-bound MHC class I chain-related (MIC) has been shown to stimulate NK cell-mediated cytokine production [30–35]. The shedding of a soluble form of this molecule (sMIC) has been shown to induce internalization of the NKG2D receptor in NK cells and consequently modulate immune responses in various pathological settings [35–37]. Endothelial expression of MIC has also been shown to target antibody-mediated vascular rejection after solid organ transplantation [38, 39]. The release of sMIC was also suggested to modulate NK cell function during pregnancy [40]. We showed that elevated sMIC serum levels, detected in the serum of 38% of infertile women candidate to in vitro fertilization, were predictive of both embryo implantation failure and pregnancy success following IVF [41].

In the present study, we first investigated whether sMIC could be detected in the plasma from women at time of VPD diagnostic. We then tested whether incubation with plasma from women with VPD could affect NKG2D expression and cell mediated production of IFN-*γ*, a major cytokine implicated in vascular modeling during pregnancy.

## 2. Subjects and Methods

### 2.1. Patients

The study population comprised 169 women with singleton gestations recruited between June 2004 and May 2006 within women attending gynecology units of Assistance Publique Hopitaux de Marseille (AP-HM, hopital de la Conception and hopital Nord, Marseille, France). All these women gave their informed consent to collect plasma and participate in the study, which was approved by the local Hospital ethics committee of our institution (CPP-Marseille 1 n°05/33).

The study population included 81 patients with a diagnostic of vascular pregnancy diseases (VPD) that were further subdivided in 3 groups: 40 patients with PE, 23 patients with IUGR, 18 patients with IUFD, 25 patients with a diagnostic of non-vascular IUGR and 63 term-matched normal pregnancies (NP control group).

The study population included 81 patients with a diagnostic of vascular pregnancy diseases (VPD) that were further subdivided in 3 groups: 40 patients with PE, 23 patients with IUGR, 18 patients with IUFD, 25 patients with a diagnostic of non-vascular IUGR and 63 term-matched normal pregnancies (NP control group).

PE was defined by persistent diastolic arterial blood pressure greater than 90 mm Hg and a systolic blood pressure greater than 140 mm Hg, associated with proteinuria (≥300 mg in a 24-hour urine collection or at least one dipstick measurement ≥2+). Severity of preeclampsia was defined according to ACOG guidelines [42], by high blood pressure ≥160 mm Hg systolic or to 110 mm Hg diastolic or presence of thrombocytopenia (platelet count less than 100.000/*μ*L) or renal failure (serum creatinine concentration greater than 1.1 mg/dL or a doubling of the serum creatinine concentration in the absence of other renal diseases), impaired liver function indicated by abnormally elevated blood concentrations of liver enzymes (2-fold normal concentration). Patients included in this study did not present other signs of severe PE such as pulmonary edema or brain or visual disturbances. Vascular IUGR was defined as ultrasonographic measurement <2.5th percentile for gestational age and baby birth weight <5th percentile associated with at least one biological or sonographical marker of “placental insufficiency” as abnormal uterine, or umbilical artery Doppler, or elevated plasma fibronectin level. Exclusion criteria of vascular IUGR group were the presence of congenital malformations or chromosomal abnormalities in the fetus, recent cytomegalovirus or toxoplasma infection, trauma, drugs or alcohol abuse during pregnancy.

The nonvascular IUGR group consisted of 25 women with isolated IUGR, related to chromosomal fetal aneuploidy in 4 cases, fetal polymalformative syndrome in 8 cases, toxic or infectious origin in 3 cases, and fetus small for gestational age in the 10 remaining cases.

Nonvascular IUGR was diagnosed as ultrasonographic measurement <2.5th percentile for gestational age and baby birth weight <5th percentile with a normal fibronectin level, uterine and umbilical artery Doppler velocity.

IUFD was defined by ultrasound examination as a visible fetus without cardiac activity after 12 weeks of gestation. Only IUFD occurring after severe vascular IUGR were included in the study. The term at sampling was the term of IUFD diagnosis (Table 1).

The control group consisted of 63 healthy pregnant women, seen for routine gynecologic examination and followed until delivery to confirm normal pregnancy (NP) outcome. Normal pregnancies were recruited between 17 and 41 weeks of gestation and matched on the pregnancy term of at least one patient of the distinct VPD groups analyzed (PE, IUGR, and IUDF).

### 2.2. Plasma Collection

Blood samples were collected at time of diagnosis of vascular pregnancies diseases or isolated IUGR and at time of obstetrical examination for the control group of term matched normal pregnancies. Samples were collected into 0.129 mol/L sodium citrate (3.8%) centrifuged and stored at −80° according to standard procedures.

### 2.3. Isolation of PBMCs

Peripheral blood from nonpregnant healthy volunteer blood donors was collected and PBMCs used for in vitro experiments were separated by Ficoll-Hypaque density gradient centrifugation.

### 2.4. Capture ELISA for sMIC/B

Soluble MIC concentrations were measured in the plasma using a sandwich enzyme-linked immunoabsorbent assay as previously described [37]. The detection threshold of recombinant soluble MICA protein, used as standard in each experiment, was 0.1 ng/mL and plasma levels higher than 0.3 ng/mL were considered as positive. Human recombinant MIC proteins standards were purified from baculovirus, as previously described [43].

### 2.5. Analysis of Cell Surface NKG2D Expression

Flow cytometry analysis of NKG2D expression was conducted after incubating control PBMCs from nonpregnant donors with 20% of human serum obtained from VPD patients or normal pregnancy controls. Intensity of PE-labeled anti-NKG2D mAb (IgG1, ON72, Beckman-Coulter) or isotype control mouse IgG1 antibody staining (Beckman-Coulter) was expressed as % of cells and mean fluorescence intensity (mfi) of NKG2D positive cells within the CD3^−^CD56^+^ peripheral blood NK cell subset. PBMCs were incubated for 48 h with 20% of sMIC-positive plasma from 8 VPD patients and analyzed in reference to sMIC-negative plasma from 8 women with normal term matched pregnancies.

## 3. Interferon-*γ* Assay Using Quantitative Real-Time PCR and ELISA

For the interferon-*γ* assay, 2 × 10^6^ NK-92 cells (ATCC number: CRL-2407) were incubated for 72 h in the presence of 20% MIC-positive or MIC-negative plasma. Total RNA was isolated from NK culture using RNeasy mini kits (QIAGEN Inc., Valencia, CA, USA) followed by reverse transcription using SuperScript RTII (Invitrogen Life Technologies) according to the manufacturer's protocol. The resulting cDNA was amplified with primer pairs specific for interferon-*γ* and GAPDH (forward and reverse sequences): INF-*γ* 5′-TGCAGAGCCAAATTGTCTCCTT-3′ and 5′-CATGTATTGCTTTGCGTTGGAC-3′; GAPDH 5′-GGTGGTCTCCTCTGACTTCAACA-3′ and 5′-GTTGCTGTAGCCAAATTCGTTGT-3′. Real-time PCR amplification was performed with the FastStart DNA Master^PLUS^ SYBR Green I Kit as recommended by the manufacturer, on a LightCycler Roche instrument. The cycling conditions were 10 min at 95°C (hot-start PCR), followed by 40 cycles of 5 s at 95°C (denaturation), 10 s at 62°C (annealing), and 20 s at 72°C (elongation). Melting curve analysis was then performed to check the specificity amplification. In relative quantification, reported values were expressed as the relative numbers of specific transcripts detected per 10^6^ GAPDH transcripts (2^−ΔCt^ method). All experiments were performed at least in duplicate. IFN-*γ* supernatant concentration of NK-cell line (Beckman Coulter, France) was measured using commercial ELISA according to the manufacturer's instructions. The limit of detection was 0.1 IU/mL.

### 3.1. Statistics

Analyses were performed using Prism software (GraphPad 4.0b, GraphPad, San Diego, CA) implementing the nonparametric Kruskal-Wallis test followed by the Dunn posttest to compare 3 or more continuous variables, the Mann-Whitney test to compare 2 unpaired groups, and the Wilcoxon matched pairs test for the NKG2D expression. Data were expressed as the median (25–75 percentile range) and mean (±sd), depending on the distribution. Association between categorical variables was tested after cross tabulation by the Pearson chi square (and by Fisher exact test if *n* < 5). Correlation was improved by spearman test. A 95% confidence interval (*P* < 0.05) was considered significant.

Multivariable logistic regression analysis was used to explore the relationship between sMIC and the following explanatory variables: parity, gestation, systolic and diastolic blood pressure, gestational age at sampling, thrombocytopenia, term of delivery, and baby weight at birth using SPSS v.12.0 (SPSS Inc., Chicago, IL, USA) statistical packages. A *P* value of <0.05 was considered significant.

## 4. Results

### 4.1. Increased Frequency of Plasma sMIC in Women with Vascular Pregnancy Diseases

Plasma levels of sMIC were evaluated in 3 groups of pregnant women: term-matched women undergoing normal pregnancies (NP), women with a diagnostic of VPD (PE or vascular IUGR or IUFD) or with a diagnostic of non-vascular IUGR. No significant differences were found in age, number of gestation, body mass index, and gestational age at sampling between normal pregnancies, VPD, and nonvascular IUGR. As expected the systolic and diastolic blood pressure were significantly higher in women with VPD. As expected the median of baby's birth weights and gestational age at delivery were significantly lower in the group with VPD and nonvascular IUGR compared to normal pregnancies (*P* < 0.001). The main clinical and biological characteristics of these patients are summarized in Table 1.

In the NP group, only one of the 63 women had detectable but low sMIC levels in plasma (0.5 ng/mL). However, it must be noted that this patient was followed for systemic lupus erythematosus, with normal pregnancy evolution. Of the 25 women with nonvascular IUGR, only one had detectable sMIC plasma levels (1.63 ng/mL). By contrast, sMIC molecules were detected in 26 of the 81 patients (32%) in the VPD group (*P* < 0.0001 in reference to NP). Highest prevalence of sMIC detection was associated with IUFD (44%) and vascular IUGR (39%) (Table 2). sMIC levels detected in the PE group (median, 25–75 interquartile ranges: 7.5 ng/mL, 1.37–32.69) were higher than those detected in the IUFD (2.18 ng/mL, 0.86–7.58, *P* < 0.01) and vascular IUGR (1.63 ng/mL, 0.86–5.2, *P* < 0.05) groups (Table 2).

### 4.2. The Presence of sMIC in PE Plasma Is Associated with Thrombocytopenia and High Proteinuria

**Figure 1:**
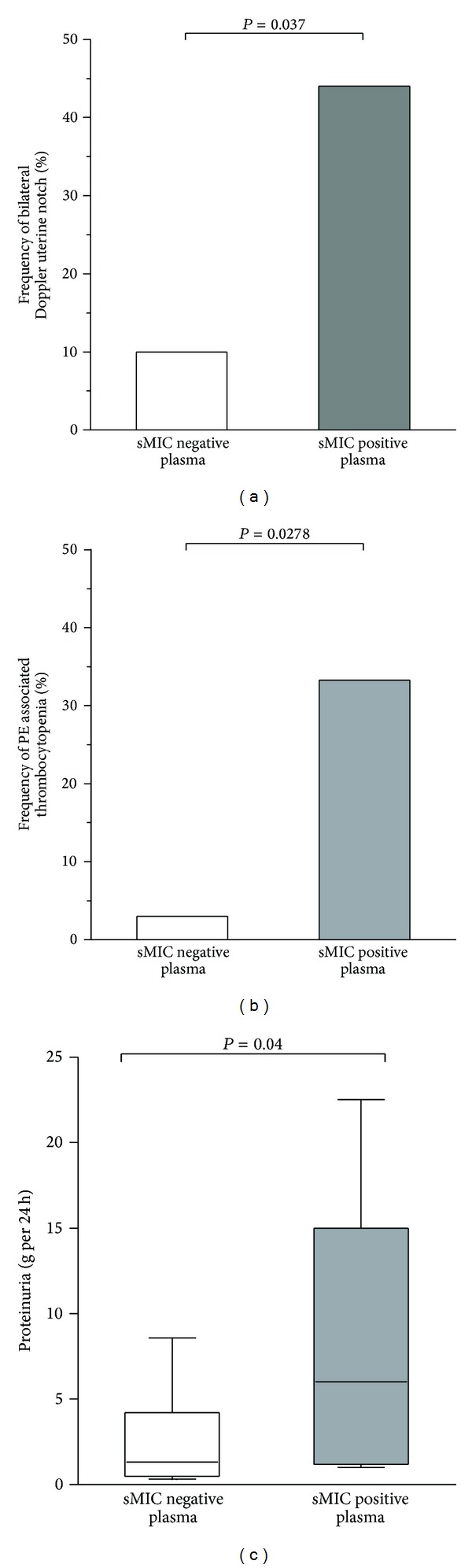
(a) Frequency of bilateral early diastolic uterine notch, in sMIC-positive and -negative subgroups of preeclamptic patients. (b) Frequency of thrombocytopenia in sMIC-positive and -negative subgroups of preeclamptic patients. (c) Median and interquartile range of proteinuria per day in sMIC-positive and -negative preeclampsia subgroups.

We then determined whether the presence of sMIC in maternal plasma could be associated with parameters that depict preeclampsia associated disorders. sMIC was detected in 22.5% of PE patients while being only observed in 1.6% of term matched normal pregnancies (*P* < 0.0008, Table 2). Sonographical analysis of maternal uterine artery Doppler velocity waveform performed at time of PE showed that bilateral early diastolic uterine notch, a reflection of placental insufficiency, was more frequent in the sMIC-positive group of PE (*P* = 0.037, Figure 1(a)). Severe PE diagnostic criteria such as high blood pressure (≥ to 160 mm Hg systolic), thrombocytopenia, or renal failure were observed in 75% of the PE cases evaluated. Among MIC^+^ patients, 89% presented criteria of PE severity: 7 patients with high blood pressure (≥ to 160 mm Hg systolic) and one patient with renal failure. In the MIC-negative group, 64% of patients were defined as severe cases of PE on the basis of hypertension or renal failure criteria. Thrombocytopenia was significantly more frequent in the sMIC-positive patients group, compared with the sMIC-negative patients group (3 to 9 patients (33%) versus 1 to 31 patients (3.0%), resp., *P* = 0.03, Figure 1(b)).

We could also show that median proteinuria per day was significantly higher in PE women with sMIC-positive plasma than in PE women without detectable sMIC (*P* = 0.04, Figure 1(c)). Multivariate analysis of parity, gestation, systolic and diastolic blood pressure, gestational age at sampling, thrombocytopenia, PAS ≥ to 160 mm Hg systolic, PAD ≥ to 110 mm Hg diastolic, term of delivery, and baby weight at birth further confirmed that proteinuria was an independent factor associated with detection of sMIC in plasma (*P* = 0.03).

### 4.3. The Presence of sMIC in Plasma Is Associated with Vascular IUGR

To further address whether the presence of plasma sMIC could be associated with the vascular origin of IUGR, we compared sMIC levels in women with vascular and nonvascular IUGR. Among the 23 patients with vascular IUGR, 9 (39%) had detectable plasma sMIC, whereas sMIC could be detected in one out of the 25 (4%) nonvascular IUGR cases. Thus while age, term at sampling, baby birth weight, and term of delivery were not different between the 2 groups, the presence of sMIC in maternal plasma was significantly associated with the vascular etiology of IUGR (*P* = 0.0038). Furthermore, analysis of the 48 pregnancies at IUGR diagnostic showed that detection of plasmatic sMIC levels in plasma is a specific factor associated with vascular etiology of IUGR, (96% specificity and 39% sensitivity).

### 4.4. Downmodulation of NKG2D Expression at the Surface of NK Cells by sMIC-Positive Plasma from VPD Patients

**Figure 2:**
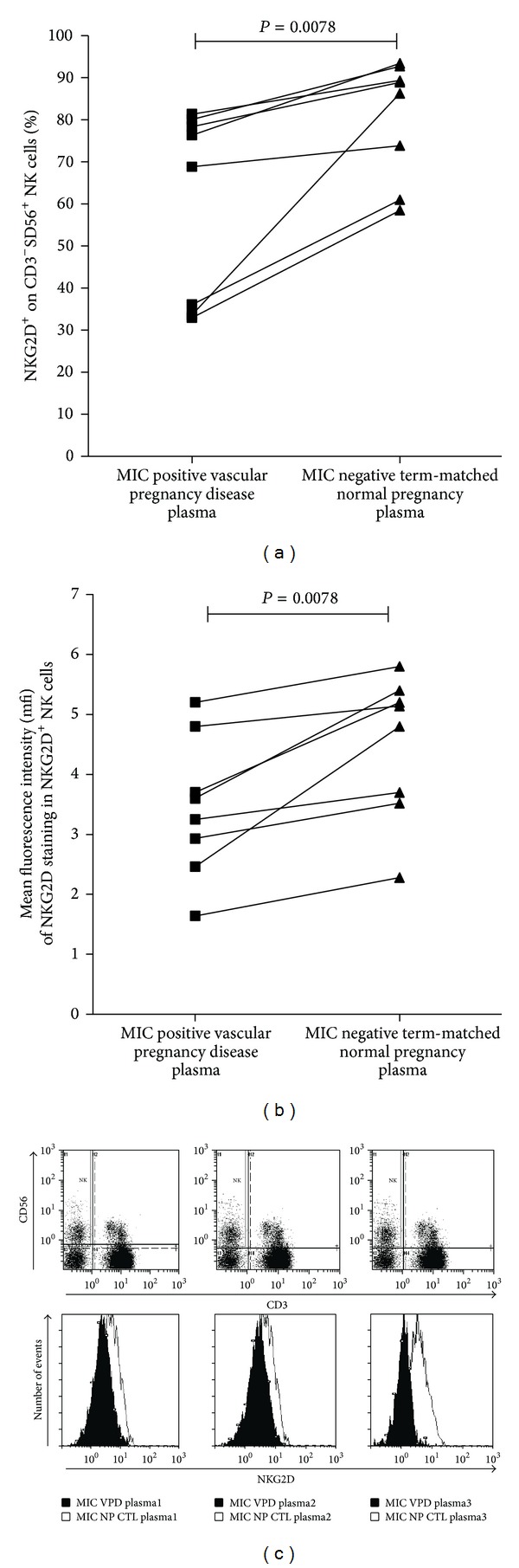
Differential effect of plasma isolated from sMIC-positive VPD patients or term-matched sMIC-negative plasma of women undergoing normal pregnancy (NP) on percentage (Figure 2(a)) and mean fluorescence intensity (mfi, Figure 2(b)) of NK cell surface NKG2D expression within PBMC. For each experiment, PBMC isolated from healthy nonpregnant control donors (NP) was cultured for 48 hours before flow cytometry analysis in media containing either 20% sMIC-positive plasma from women experiencing vascular pregnancy diseases (black) or 20% sMIC-negative plasma from normal pregnancies (NP, gray). Gestational age at plasma sampling of VPD cases was matched to that of NP plasma used as control in 8 independent experiments. Plasma-induced modifications of NKG2D cell surface expression are illustrated as variation in the % of NKG2D positive CD3^−^CD56^+^ NK cells found within PBMC (a) and mean fluorescence intensity of NKG2D staining in NKG2D positive CD3^−^CD56^+^ NK cells (b). (c) Flow cytometry plots illustrate detection of NK cells and gating of NKG2D expression within CD3^−^CD56^+^ NK cells in 3 representative experiments. Overlay of NKG2D downregulation resulting from incubation of NK cells with sMIC-positive VPD plasma (black) versus sMIC-negative plasma from control normal undergoing pregnancies (NP Figure 2(c), right panel). Wilcoxon matched pairs tests were used to compare the two groups.

We then analyzed whether plasma from sMIC-positive VPD patients induced modification of NKG2D expression at the surface of NK cells within peripheral blood mononuclear cells (PBMCs) isolated from healthy donors (*n* = 8). PBMCs were incubated for 48 h with 20% of sMIC-positive plasma from 8 VPD patients and analyzed in reference to sMIC-negative plasma from 8 women with normal term matched pregnancies. Flow-cytometry analysis of NKG2D expression in CD3^−^CD56^+^ NK cells showed that incubation with sMIC-positive plasma from VPD resulted in a significant decrease in NKG2D expression in both % (Figure 2(a)) and mean fluorescence intensity (mfi, Figure 2(b)) (median, 25–75 interquartile ranges: % of NKG2D 72.25, 34.7–78.8; mfi 3.4, 2.7–4.2) when compared to sMIC-negative plasma of NP matched for term (% of NKG2D 87.2%, 67.1–90.6; MFI 5, 3.6–5.3, *P* = 0.0078). Moreover, the levels of plasma-induced downregulation of NKG2D at the surface of NK cells (Δ in % of NKG2D stained NK cells observed after incubation with MIC-positive plasma relative to MIC-negative plasma) were correlated to VPD sMIC plasma levels (Spearman correlation, *r* = 0.76, *P* = 0.04).

### 4.5. sMIC-Positive Plasma of VPD Patients Impairs NK Cell-Mediated IFN-*γ* Production

**Figure 3:**
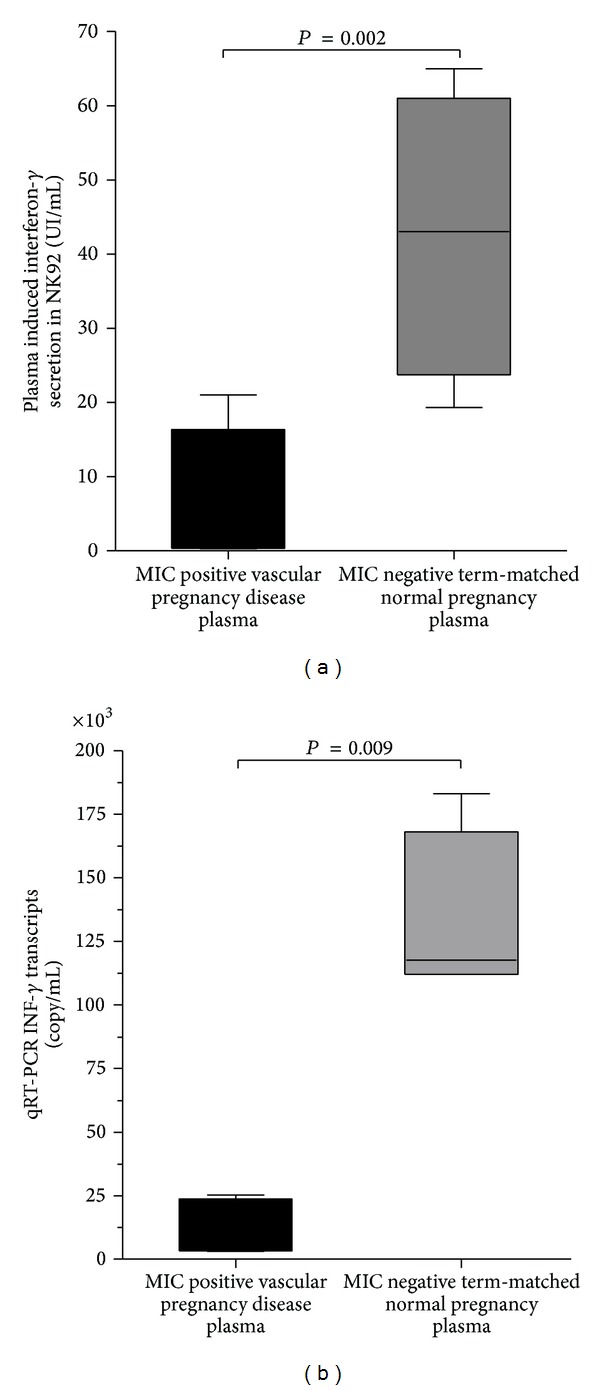
Quantification of INF-*γ* secretion by the NK-92 NK cell line after 72 hours incubation in media containing 20% plasma from representative sMIC-positive vascular pregnancy diseases (VPD, sMIC^+^) and gestational age-matched sMIC-negative plasma normal pregnancies (NP, sMIC^−^). (a) ELISA detection of INF-*γ* levels in supernatants of NK-92 cells cultured in 20% plasma. (b) Real-time PCR transcriptional analysis of INF-*γ* mRNA levels in NK-92 cells. mRNA transcript levels are represented as relative copy numbers per 10^6^ GAPDH transcripts. Results are expressed as median and 25–75 interquartile ranges (*n* = 4).

To gain insight into the mechanisms by which sMIC could affect NK-cell dependent vascular placental remodeling, we then investigated whether sMIC-positive VPD plasma could modulate NK cell-mediated INF-*γ* production. NK-92 cell lines were cultured for 72 hours in medium containing either 20% sMIC-positive plasma from VPD patients or 20% sMIC-negative plasma of term-matched women undergoing normal pregnancies. Impact of plasma was evaluated by ELISA assay of IFN-*γ* secretion levels in NK cell supernatants and real-time qRT-PCR analysis of INF-*γ* mRNA transcript levels in NK cells. We showed that INF-*γ* levels in culture supernatants were significantly reduced (15-fold decrease) after incubation of NK cells with sMIC-positive VPD plasma (median, 25–75 interquartile range: 2.5 ng/mL, 0.42–14.4), when compared to sMIC-negative NP plasma (41 ng/mL, 28.1–57 *P* = 0.002, Figure 3(a)). Furthermore, when analyzed by quantitative RT-PCR, relative levels of IFN-*γ* transcripts were also significantly decreased in the presence of sMIC-positive VPD plasma (median values relative to GAPDH, 25–75 interquartile range: 19170, 3140–25295), as compared to sMIC-negative NP plasma (123279, 112000–183011, *P* = 0.04, Figure 3(b)).

## 5. Discussion

Identification of specific biomarkers that characterize VPD, whose values may differ from observations occurring outside of the pregnancy context, is a challenging issue to anticipate adverse outcomes of these diseases for the mother and the child. Hypertensive disorders of pregnancy are a major factor associated with 5–10% of pregnancies and represent the second commonest cause of direct maternal death [44]. Gestational hypertension, renal failure, and congenital thrombophilic defects represent major criteria of PE diagnostic and have been associated with the occurrence and severity of PE [45, 46]. While standard diagnostic criteria have evolved, PE remains an extremely heterogeneous disease reflecting interplay of multiple underlying processes that can vary from one woman to another. It is thus important to unravel mechanisms and molecules that may reflect the heterogeneity of the clinical syndromes and enhance our understanding of how they relate to adverse pregnancy outcome in one woman and not in the other.

Deregulated expression profiles of a wide array of vascular and thrombotic markers have been identified to be associated with the onset and severity of vascular disorders that complicate pregnancies [5, 6, 47, 48]. Apart from angiogenic factors* per se*, the relevance of immunological markers has also been highlighted in vascular disorders of pregnancy and notably in the multisystem disorders associated with preeclampsia. The primary defect that leads to preeclampsia indeed involves alteration in the dialogue between invasive placental trophoblast cells and maternal immune cells in the uterine wall. Extravillous trophoblast cells express an unusual combination of polymorphic and nonpolymorphic HLA class I molecules that impact uterine NK-cell (uNK) angiogenic function during pregnancy. Genetic and functional studies suggested that maternal KIR/fetal HLA-C and G interactions regulate the delivery of an optimal blood supply to mother and fetus. The adverse impact of genetic KIR/HLA-C combinations on trophoblast-cell invasion during PE has been reported, as a support for the diversity of maternal immune responses during placentation [3].

Our study provides the first evidence that detection of soluble MIC-A, a stress inducible immunomodulatory molecule, is more frequent in plasma from women with vascular pregnancy complications than in gestational age-matched normal pregnancies or nonvascular IUGR.

Considering the result of this study, we expect that sMIC could be a stress marker reflecting disorders of the maternal immune system that impact NK cell angiogenic function at the fetomaternal interface in some women. As previously described for women that experience fetal implantation failure after IVF, plasmatic levels of maternal sMIC are only detected in about one third of PVG cases, with higher prevalence in women that experience intrauterine fetal death after IUGR or with a diagnostic of vascular intrauterine growth retardation or PE. This suggests that the level of sMIC upregulation in maternal serum may vary in response to stress signals provided by the pathological environment during complicated pregnancies. Although the nature of these signals is presently unknown, various viral, hormonal, and environmental stimuli have been reported to induce NKG2D ligand expression [49, 50] and may influence MIC-driven modulation angiogenic function in VPD. Although the number of PVG cases evaluated in this preliminary study may be too limited to address this point, no direct association was made between sMIC levels and diastolic or systolic blood pressure measurement in the PE subgroup. In PE patients, we suggest that high levels of sMIC could rather be associated with thrombocytopenia and high proteinuria, a former diagnostic criterion of severity of PE that is no longer retained in the American College of Obstetricians and Gynecologists' 2013 guidelines. Of interest, our results suggest that presence of sMIC in maternal plasma was also associated with the vascular nature of pregnancy-related disorders that lead to IUGR. IUGR is a pregnancy complication with many etiologies that associate to the highly variable prognosis of this disease. Placental insufficiency constitutes the major cause of vascular IUGR. Most vascular IUGR share the physiopathology signs of PE [51], and vascular IUGR is sometimes diagnosed in the presence of PE or alteration of uterine Doppler Velocimetry wave flow. By contrast, isolated IUGR may represent the only sonographical sign of severe fetal infection, aneuploidy, or genetic syndrome associated with abnormal neurologic outcomes. However, there is a lack of noninvasive and specific markers that allow differentiating vascular from nonvascular IUGR. Indices of uterine Doppler Velocimetry, known as the best marker in the prediction of placental hypoxic-ischemic lesions in IUGR, show 63% specificity for 97% sensibility [52]. In the absence of obvious IUGR etiology, invasive fetal explorations are required to determine fetal prognosis, with nonnegligible rate of premature delivery and late fetal loss [53]. Our observation that sMIC detection in plasma of women who developed IUGR may discriminate the vascular origin of IUGR with 96% specificity prompts further larger scale prospective studies that may confirm the value of sMIC to characterize or anticipate the vascular etiology of IUGR.

Although the mechanisms by which sMIC may participate to vascular pregnancy diseases remain unclear, we bring evidence that sMIC-containing plasma from patients with vascular pregnancy disorders can impact NKG2D expression and modulate NK-mediated IFN-*γ* production. Although factors that impair NK-mediated IFN-*γ* production have been suggested to impact vascular remodeling, their alteration in association with vascular disorders of pregnancy is still poorly documented. Recent findings indicate that IFN-*γ*-secreting NK cells play a proangiogenic role and promote VEGF expression and corneal neovascularization [54, 55]. Several reports highlighted the role of dNK-derived INF-*γ* as a regulator of the size of uterine blood vessels that favors adequate decidualisation in a murine model [55–57]. In humans, various studies bring evidence for a direct role for dNK in modulating extravillous trophoblast cell differentiation and migration from anchoring villi, which is in part dependent from their potential to deliver IFN-*γ* [58]. Impaired NK cell mediated release of IFN-*γ* has been recently reported in women with hypertensive disorders that complicate pregnancies [15]. Integration of microenvironment signals delivered to NK cells at the fetomaternal interface is expected to affect this essential process of successful pregnancy. Fine tuning of NK-mediated cell cytokine production is dependent on engagement of cell surface receptors that sense HLA ligand expression on trophoblast cells. The high level of diversity of receptors and ligand that may regulate these interactions may in part condition the efficiency of placentation [59]. Genomic evidence suggests that deleterious combinations of maternal NK cell receptor repertoire and fetal HLA-C and HLA-G antigens can shape a strong inhibitory NK phenotype associated with impaired vascular placental remodeling [60]. Altered expression of the HLA-G NK cell ligand has also been reported to have a significant influence on IFN-*γ* production by NK cells [21] and development of preeclampsia [61, 62]. Consistent with this view, lowered levels of soluble HLA-G protein in maternal plasma were associated with occurrence of severe PE and IUGR during the third trimester [28, 63]. Similarly, we can speculate that stress-induced upregulation of the highly polymorphic MIC molecule may also differentially impact essential immune functions during placentation. Our results indeed suggest that presence of sMIC in plasma of women with vascular pregnancy diseases can deliver a strong inhibitory signal to NK cells through downmodulation of cognate NKG2D receptor expression at the surface of NK cells and NK-cell interferon-*γ* secretion. We have recently shown that detection of sMIC levels in the serum of some infertile women is predictive of implantation failure and successful term pregnancy following in vitro fertilization [41]. These data suggest that deregulated expression of this stress-inducible immunomodulatory protein may be associated with immune and vascular dysfunction during pregnancy, notably by impairing NK-mediated production of IFN-*γ*, an essential cytokine to allowing implantation and placentation.

Results from this exploratory study thus suggest that noninvasive evaluation of sMIC may be a hallmark of underlying mechanisms that complicate pregnancies in some women. It prompts larger prospective studies that may allow determining how a stress-inducible immunomodulatory molecule reflecting immune disorders may relate to other markers defining “angiogenic” and “nonangiogenic” profiles associated with pregnancy outcome.

In summary, we bring the first evidence that detection of high sMIC plasma levels may constitute a noninvasive indicator of underlying immune-mediated disorders of placentation in some women with severe VPD and may notably characterize the vascular etiology of IUGR. We further suggest that, in the pathogenic context of some pregnancies, sMIC release may constitute a factor that impairs immune cell's potential to establish the proper context of vascular remodeling steps, thus favoring the onset of severe vascular disorders that affect pregnancy.

## Figures and Tables

**Table 1:** Characteristics of study population.

	Normal pregnancies	Vascular pregnancy diseases	Nonvascular IUGR	*P* value
Number of patients	63	81	25	
Age (yrs, mean ± sd)	29.6 ± 6.6	30.2 ± 6.3	29 ± 6.3	ns
Gestation (*n*)	3 [1–9]	2 [0–7]	2 [0–6]	ns
Parity (*n*)	1 [0–5]	0 [0–4]	0 [0–3]	ns
Body mass index (kg/cm^2^)	23 [17–48]	23.1 [16.3–37.2]	23 [18–37]	ns
Systolic blood pressure (mmHg)	12 [10–13]	14 [10–22]	12 [10–13]	*P* < 0.001*
Diastolic blood pressure (mmHg)	7 [5–8.3]	8.7 [6–12]	7 [5.5–8.5]	*P* < 0.001*
Uterine height (cm)	32 [29–38]	26 [15–36]	26 [15–36]	*P* < 0.001^§^
Term at sampling (weeks of gestation)	31.7 [15.3–41]	30 [17–41]	30 [17–41]	ns
Term at delivery (weeks of gestation)	40.3 [35–42]	32.3 [17–41.1]	32 [22–41]	*P* < 0.001^§^
Baby weight at birth (g)	3300 [2640–4680]	1330 [80–3410]	1745 [400–2500]	*P* < 0.001^§^

Comparison between groups was performed with nonparametric Kruskal-Wallis test followed by the Dunn posttest. Values indicate median [25–75 interquartile ranges].

*P < 0.001 between vascular pregnancy diseases and other groups.

^§^ *P* < 0.001 between normal pregnancies and other groups.

**Table 2:** Frequency of sMIC detection and plasma levels in the study population.

	Frequency of sMIC positive plasma number (proportion %)	Comparison of sMIC frequency in reference to control normal pregnancy group	Median sMIC plasma levels in positive samples (ng/mL) [25–75 interquartile range]
Normal pregnancies *n* = 63	1 (1.6%)	—	0.5
Vascular pregnancy diseases *n* = 81	26 (32%)	*P* < 0.0001	2.2 [1.15–11.47]
Preeclampsia *n* = 40	9 (22.5%)	*P* < 0.0008	7.5 [1.37–32.69]
Vascular IUGR *n* = 23	9 (39%)	*P* < 0.0001	1.63 [0.86–5.2]
IUFD *n* = 18	8 (44%)	*P* < 0.0001	2.18 [0.86–7.58]
Nonvascular IUGR *n* = 25	1 (4%)	ns	1.63
